# Modulating β-catenin/BCL9 interaction with cell-membrane-camouflaged carnosic acid to inhibit Wnt pathway and enhance tumor immune response

**DOI:** 10.3389/fimmu.2023.1274223

**Published:** 2023-10-09

**Authors:** Ruqing Gao, Xiaoqiang Zheng, Aimin Jiang, Wangxiao He, Tianya Liu

**Affiliations:** ^1^ Department of Medical Oncology, The First Affiliated Hospital of Xi’an Jiaotong University, Xi’an, China; ^2^ School of Medicine, Nanchang University, Nanchang, China; ^3^ Institute for Stem Cell & Regenerative Medicine, The Second Affiliated Hospital of Xi’an Jiaotong University, Xi’an, China; ^4^ Department of Talent Highland, The First Affiliated Hospital of Xi’an Jiaotong University, Xi’an, China

**Keywords:** Wnt/β-catenin signal, lung adenocarcinoma, Wnt pathway suppressor, carnosic acid, cancer therapy, tumor immune response

## Abstract

**Introduction:**

Lung adenocarcinoma (LUAD) therapies are plagued by insufficient immune infiltration and suboptimal immune responses in patients, which are closely associated with the hyperactive Wnt/β-catenin pathway. Suppressing this signaling holds considerable promise as a potential tumor therapy for LUAD, but Wnt suppressor development is hindered by concerns regarding toxicity and adverse effects due to insufficient targeting of tumors.

**Methods:**

We have synthesized a tumor-specific biomimetic Wnt pathway suppressor, namely CM-CA, by encapsulating carnosic acid within Lewis lung carcinoma (LLC) cell membranes. It possesses nano-size, allowing for a straightforward preparation process, and exhibits the ability to selectively target the Wnt/β-catenin pathway in lung adenocarcinoma cells. To evaluate its *in vivo* efficacy, we utilized the LLC Lewis homograft model, and further validated its mechanism of action through immunohistochemistry staining and transcriptome sequencing analyses.

**Results:**

The findings from the animal experiments demonstrated that CM-CA effectively suppressed the Wnt/β-catenin signaling pathway and impeded cellular proliferation, leading to notable tumor growth inhibition in a biologically benign manner. Transcriptome sequencing analyses revealed that CM-CA promoted T cell infiltration and bolstered the immune response within tumor tissues.

**Conclusion:**

The utilization of CM-CA presents a novel and auspicious approach to achieve tumor suppression and augment the therapeutic response rate in LUAD, while also offering a strategy for the development of Wnt/β-catenin inhibitors with biosafety profile.

## 1 Introduction

Lung cancer stands as a leading contributor to global mortality rates and occupies a prominent position in terms of cancer occurrence and fatality in China, of which approximately 85% of patients are diagnosed with non-small cell lung cancer (NSCLC) (1–3). Among NSCLC subtypes, lung adenocarcinoma emerges as the prevailing form accounting for approximately 40% of cases, and is associated with an unfavorable prognosis (4, 5). Despite the advent of novel treatments like immunotherapy, which have demonstrated favorable outcomes in select individuals, the present median overall survival (OS) for patients with metastatic NSCLC remains below three years due to the insufficient tumor immune infiltration and suboptimal immune response (4, 6, 7). Among the signaling pathways associated with lung cancer, the excessive activation of the Wnt/β-catenin pathway has garnered significant interest. This aberrant activation leads to the up-regulation of proteins related to the cell cycle, promotion of tumor cell proliferation, enhancement of intratumoral vascular density and maintenance of tumor stem cell stemness, thereby greatly contributing to the processes of tumorigenesis, progression, and metastasis (8–11). There is a growing amount of evidence indicating that the aberrantly hyperactive Wnt/β-catenin signaling significantly impedes various steps of the tumor immune response, particularly the activation and infiltration of T cells, and hence plays a pivotal role in facilitating the immune evasion of tumor cells (12–16). Consequently, targeting the inhibition of Wnt/β-catenin signaling holds promise for yielding diverse anti-tumor effects and possesses substantial therapeutic prospects.

Encouraging progress has been made in utilizing Wnt/β-catenin pathway inhibition as a potential therapeutic approach against tumors: inhibition targets encompass both upstream components and downstream target genes; various types of agents, including small molecules, peptides, and antibodies, have been investigated; and numerous studies have been conducted, ranging from preclinical animal models to clinical trials (17–22). Nevertheless, the development of Wnt inhibitors continues to pose considerable challenges. Primarily, the selection of an appropriate target for inhibition necessitates careful consideration. The Wnt/β-catenin pathway is intricately interconnected, with a diverse array of downstream target genes and complicated crosstalk between its various components and other pathways (23–25). It means that inhibiting upstream components may result in the disruption or deregulation of other normal pathways, while solely targeting downstream ones may prove inadequate in demonstrating anti-tumor effects at the cellular or tissue level. Furthermore, the Wnt/β-catenin pathway plays a crucial role in various cellular physiological processes both in normal and tumor cells, which necessitating a concentrated effort on addressing the toxicity and adverse effects associated with inadequate tumor targeting of inhibitors (26, 27). This is also the reason why numerous candidate inhibitor molecules failed to translate their promising *in vitro* results to *in vivo* efficacy. As a result of these significant constraints, there are currently no approved drugs specifically targeting the Wnt/β-catenin pathway for safe clinical utilization.

**Figure 1:**
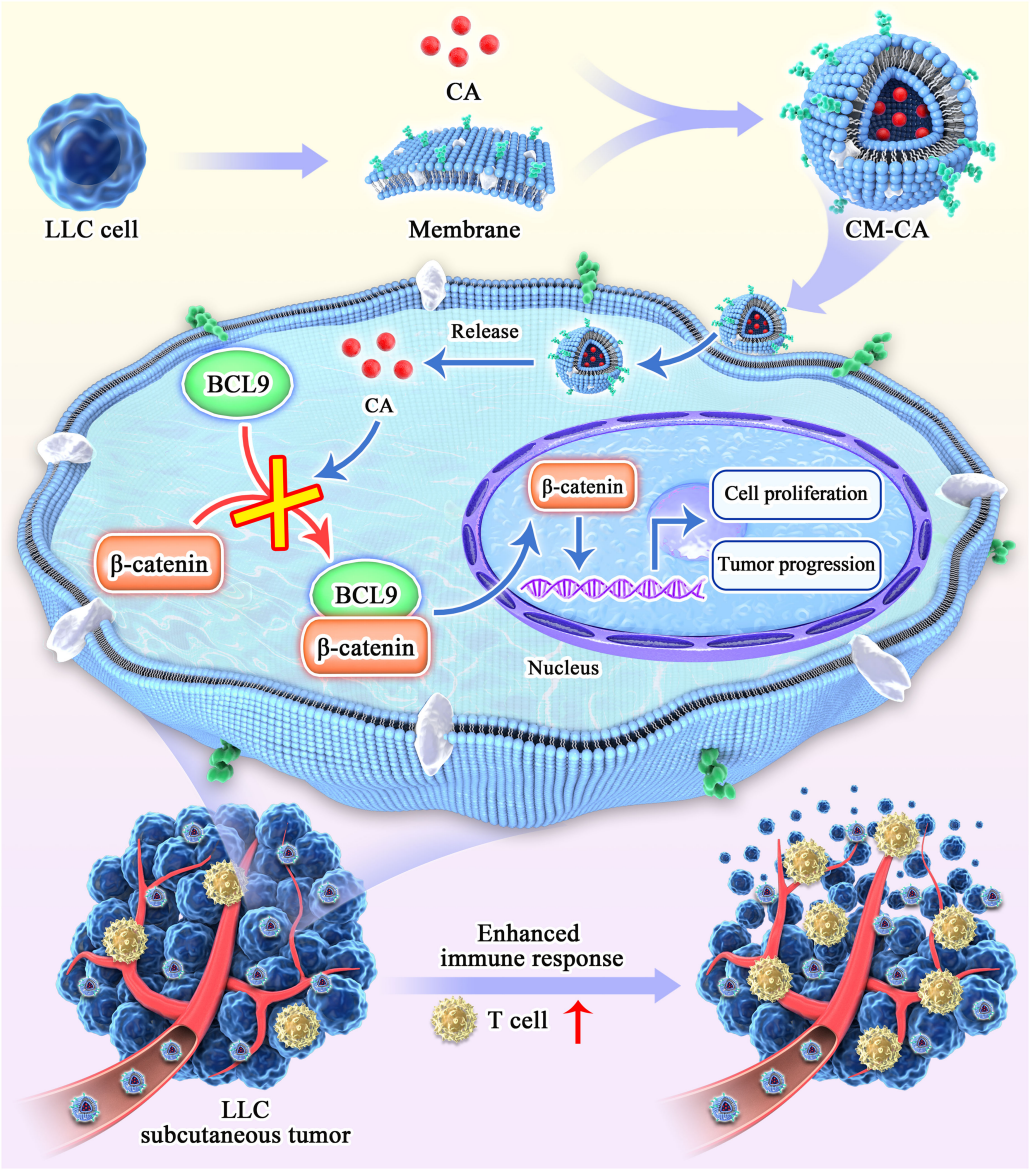
Schematic diagram of the synthesis and action process of CM-CA. CM-CA was acquired through the encapsulation of CA within LLC cell membranes. Upon CM-CA reaching tumor tissues, CA was subsequently released within tumor cells, impeding the β-catenin/BCL9 binding. This inhibition effectively curtailed the over-activation of the Wnt/β-catenin pathway within tumor cells, leading to a noticeable augmentation in T-cell infiltration and an enhanced immune response within the tumor tissues.

In order to tackle the aforementioned concerns, here, we developed a tumor-specific biomimetic Wnt pathway suppressor with nano-size and a convenient preparation process, capable of targeting the Wnt/β-catenin pathway in lung adenocarcinoma cells. Our choice of β-catenin as the function target is based on its pivotal role within the signaling cascade, ensuring specific and potent suppression of the canonical Wnt pathway (28, 29). β-catenin undergoes translocation from the cytoplasm to the nucleus to initiate transcription of downstream target genes, of which the transcription activation is facilitated by the BCL9 protein through direct binding with β-catenin (30). The application of carnosic acid (CA), a promising molecule derived from natural extracts capable of directly disrupting the β-catenin/BCL9 interaction, is hindered by its limited *in vivo* utilization and the presence of additional toxic side effects (31, 32). To improve tumor targeting specificity and biocompatibility, cell membranes from Lewis lung carcinoma (LLC) cells were employed to encapsulate CA molecules, resulting in the formation of nano-sized biomimetic particles (named CM-CA). CM-CA is designed not only to benefit from the passive targeting advantages achieved through the enhanced permeation and retention (EPR) effect, but also to possess the ability of recognition and acceptance by syngeneic tumor cells as well as to evade immune clearance, thereby achieving tumor-specific Wnt/β-catenin pathway suppression. The results of animal experiments indicate that CM-CA possesses a greater *in vivo* inhibitory effect on β-catenin compared to CA. Additionally, CM-CA promotes tumor immune infiltration and enhances the immune response against tumors, resulting in a more pronounced suppression of tumor growth. (**Figure 1**) Our work offers a novel candidate inhibitor for targeting the Wnt/β-catenin pathway in lung cancer, and presents an approach to developing Wnt inhibitors that effectively balance safety and efficacy.

## 2 Results

### 2.1 Increased expression of β-catenin is correlated with immunosuppressive microenvironment, promoting tumor progression and metastasis in lung adenocarcinoma

**Figure 2:**
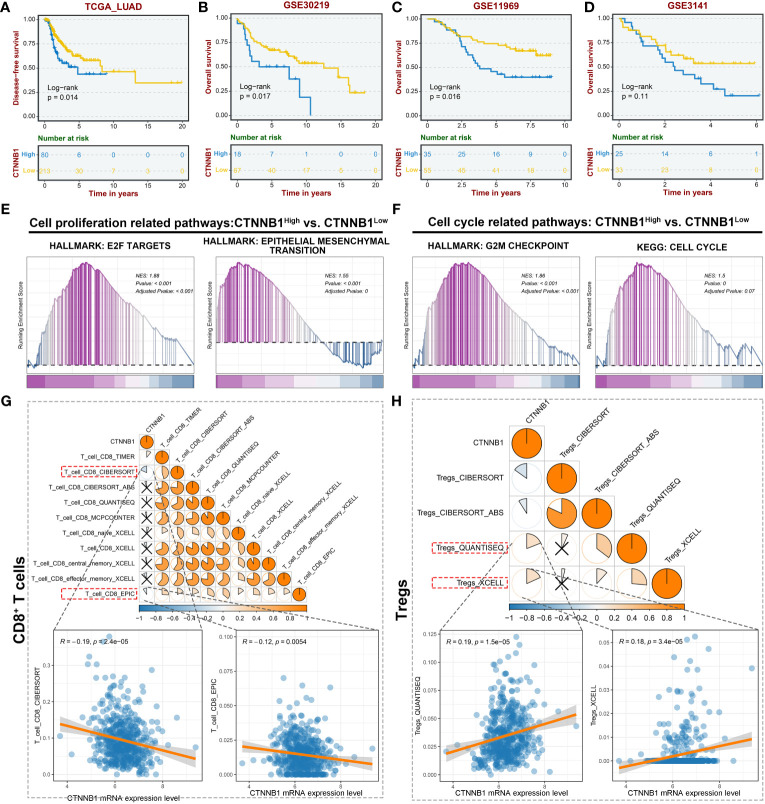
CTNNB1 is correlated with inferior prognosis and immunologic characteristics of the malignant phenotype in LUAD. **(A–D)** Kaplan-Meier survival curves to demonstrate the survival difference between high and low CTNNB1 groups in multiple LUAD datasets, including TCGA-LUAD cohort **(A)**, GSE30219 cohort **(B)**, GSE11969 cohort **(C)**, and GSE3141 cohort **(D)**. **(E, F)** GSEA revealing the alterations in the cell proliferation related pathways **(E)** and cell cycle related pathways **(F)** between the high and low CTNNB1 groups. **(G)** The correlation between CTNNB1 mRNA expression level and CD8^+^ T cells infiltration level in the TCGA-LUAD cohort. The upper panel is the correlation estimated using multiple algorithms. The lower panels are the correlation scatter plots estimated by CIBERSORT and EPIC. **(H)** The correlation between CTNNB1 mRNA expression level and Tregs infiltration level in the TCGA-LUAD cohort. The upper panel is the correlation estimated using multiple algorithms. The lower panels are the correlation scatter plots estimated by QUANTISEQ and XCELL.

To determine the potential impact of CTNNB1(encoding β-catenin) expression on the survival of patients with lung adenocarcinoma, an analysis was conducted using data from The Cancer Genome Atlas Program (TCGA). A cohort of 293 lung adenocarcinoma (LUAD) patients was categorized into two groups, the high-CTNNB1 and low-CTNNB1 groups, based on the expression level. According to the data presented in **Figure 2A**, a notable disparity in survival rates was observed between the two groups. Specifically, patients exhibiting higher β-catenin expression demonstrated significantly poorer survival outcomes compared to those with low expression, which is further substantiated by the Kaplan-Meier survival curves for the high and low CTNNB1 expression groups in the remaining three cohorts of human lung adenocarcinoma samples (33–36). (**Figures 2B–D**) Additionally, we found that the high-CTNNB1-expression group exhibited an enrichment of activation signals pertaining to tumor cell proliferation and metastasis-related pathways (E2F targets, Epithelial-mesenchymal transition). (**Figure 2E**) Simultaneously, in contrast to patients exhibiting low expression of CTNNB1, the high expression group demonstrated an up-regulation of cell cycle related pathways (G2M Checkpoint, Cell Cycle). (**Figure 2F**) These findings indicate that the hyperactive β-catenin, the pivotal constituent of the Wnt/β-catenin pathway, may facilitate tumor cell proliferation, metastasis and invasion in LUAD.

In light of the strong association between the tumor immune microenvironment, tumor heterogeneity, and tumor progression in LUAD (37), we conducted an analysis to examine the relationship between β-catenin expression and immune infiltration within the TCGA-LUAD cohort. The result indicated a noteworthy negative correlation between CTNNB1 mRNA expression level and CD8^+^ T cell infiltration level. (**Figure 2G**) Conversely, a positive correlation was observed between the mRNA expression level of CTNNB1 and regulatory T cells (Tregs), as confirmed by multiple algorithms. (**Figure 2H**) The dominance of CD8^+^ T cells in the anti-tumor immune response, and the role of Tregs in promoting antigenic tolerance to tumor cells potentially resulting in immune escape, are widely recognized (38, 39). These findings suggest that the expression level of β-catenin plays a crucial role in the development of the tumor immunosuppressive microenvironment. Furthermore, they offer a plausible explanation for the observed association between high β-catenin expression and poor response to LUAD immunotherapy. Collectively, targeted inhibition of excessively active β-catenin within tumor cells has the potential to yield various anti-tumor outcomes, and this renders β-catenin an auspicious therapeutic target.

### 2.2 CM-CA effectively inhibited tumor growth in LLC Lewis homograft model of lung cancer

The aforementioned impacts of the Wnt/β-catenin pathway on tumor progression, showed us the anti-tumor potential of CM-CA as a β-catenin/BCL9 interaction blocker. To assess the *in vivo* anti-tumor efficacy of CM-CA, we established an LLC Lewis homograft model of murine lung adenocarcinoma by subcutaneously injecting LLC cells (8 × 10^5^ per mouse) into C57BL/6 mice. The established model mice were randomly divided into three groups (n=5/group), treated with different substances: PBS (Control), CA, and CM-CA. Specifically, when the tumor volume reached approximately 50 mm^3^, a 14-day administration was initiated by drug injection via tail vein at a dosage of 2 mg/kg. The frequency of administration was once every two days. The animal protocol received approval from the medical ethics committee of Xi’an Jiaotong University (approval number 2020-277). The transmission electron microscopy (TEM) image, measurement of the encapsulation efficiency, particle size distribution and zeta potential data proved that the CM-CA used in the experiments possess normal particle morphology and stability with relatively satisfactory CA loading. (**Supplementary Figures S1–S5**).

**Figure 3:**
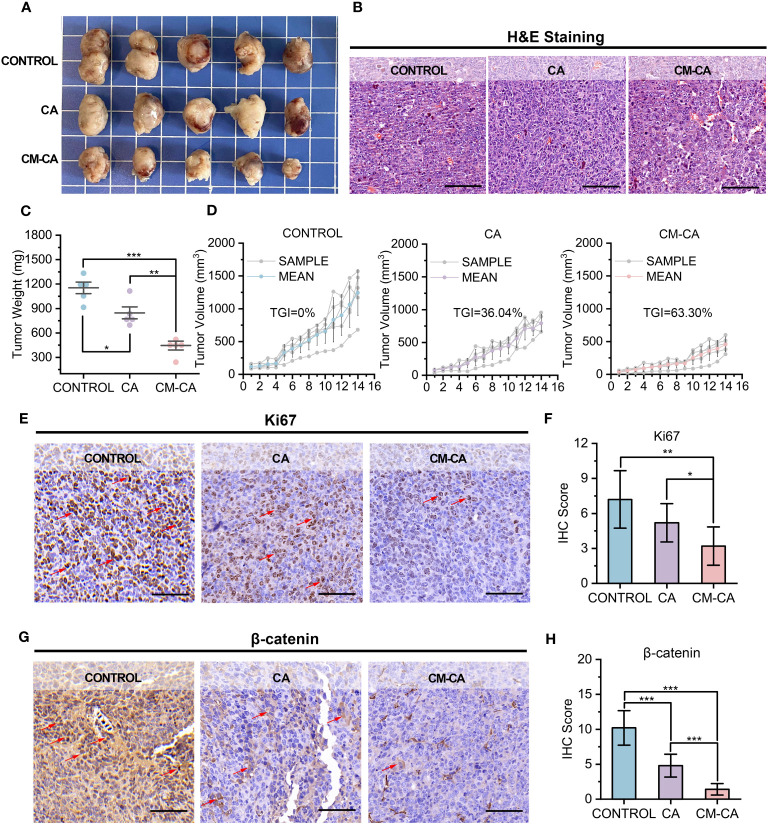
CM-CA effectively inhibited tumor growth in LLC Lewis homograft model. **(A)** Representative photographs of tumors from mice after the indicated treatments. **(B)** H&E staining of tumor sections. (scale bar: 80 µm) **(C, D)** Weights of tumors **(C)** and growth curves of tumor volumes **(D)** from mice receiving the 14-day administrations. **(E, F)** Ki67 immunohistochemical staining images **(E)** and IHC scores **(F)** of the mice tumor sections after the indicated treatments. (scale bar: 80 μm; selected area=5) The positive cells were marked by red arrows. **(G, H)** β-catenin immunohistochemical staining images **(G)** and IHC scores **(H)** of the mice tumor sections after the indicated treatments. (scale bar: 80 μm; selected area=5) The representative positive cells were marked by red arrows. The statistical difference was determined by t test. *, p<0.05; **, p<0.01; ***, p<0.001.

At the conclusion of the 14-day administration, the mice were executed and the tumor tissues were extracted for further examination. **Figure 3A** displays photographs of the tumors, which visually demonstrate that the growth of tumors in the mice treated with CM-CA was impeded. This observation is further supported by the results of hematoxylin & eosin (H&E) staining, as depicted in **Figure 3B**, which provide evidence from the perspective of tumor histopathology. In comparison to the Control and CA groups, the tumor weights of mice in the CM-CA group exhibited a remarkable reduction with a statistically significant. (**Figure 3C**) Analysis of the tumor volume curves for the three groups indicated that both CA and CM-CA treatments demonstrated varying degrees of inhibition on tumor volume growth. Notably, the CM-CA treatment exhibited a particularly significant effect, as evidenced by its average tumor volume being approximately one-third of that observed in the control group at the end of administration. (**Figure 3D**) Significantly, the tumor suppression rate observed in the CM-CA group (TGI=63.30%) was found to be approximately twice as high as that observed in the CA group (TGI=36.04%) when subjected to the same therapeutic management protocol, suggesting that CM-CA exhibited a superior efficiency in suppressing tumor growth in LLC Lewis homograft LUAD model compared to CA. The Ki67 immunohistochemical staining results of tumor tissues revealed a considerable decrease in the Ki67 expression level in tumor tissues from CM-CA-treated mice compared to mock-/CA-treated ones, indicating a notable inhibition of tumor cell proliferation. (**Figures 3E, F**) Meanwhile, we examined the β-catenin levels of tumor tissues from each group by immunohistochemistry to compare and confirm whether CM-CA achieved more effective Wnt/β-catenin signaling inhibition. As anticipated, the tumor tissues of mice treated with CM-CA showed a distinct down-regulation of β-catenin levels compared to the CA-treated and control groups. (**Figures 3G, H**) Collectively, these findings demonstrate that CM-CA, when administered at the same dosage, achieves superior β-catenin signaling suppression compared to CA. Considering the impact of CA-induced inhibition of catenin on cellular proliferation and apoptosis (40, 41), this highlights the therapeutic potential of CM-CA in controlling tumor growth *in vivo*.

### 2.3 CM-CA successfully attained inhibition of the tumor Wnt signaling pathway, augmenting intratumoral immune infiltration *in vivo*

**Figure 4:**
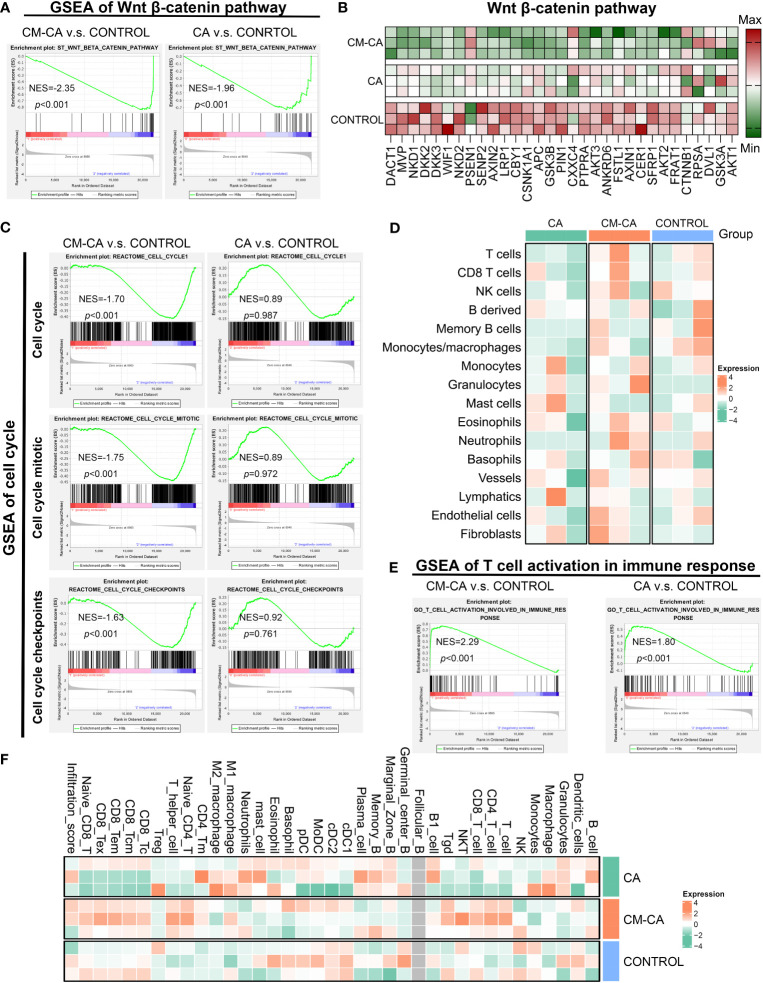
CM-CA achieved Wnt signaling pathway inhibition and augmented intratumoral immune infiltration *in vivo*. **(A)** GSEA results of Wnt β-catenin pathway in CM-CA/CA versus the control group. **(B)** Heat map of different gene expressions in Wnt β-catenin pathway among three groups. **(C)** GSEA results of cell cycle related procedures, including cell cycle, cell cycle mitotic and cell cycle checkpoints. **(D)** Heatmap plot to show the immune and stromal cells infiltration level estimated by mMCP-counter between different treatment groups. Each group has three biological replication samples. **(E)** GSEA results of T cell activation in immune response. **(F)** Heatmap plot to show the immune and stromal cells infiltration level estimated by ImmuCellAI-mouse between different treatment groups. Each group has three biological replication samples.

To elucidate the cellular and molecular mechanisms underlying the tumor growth inhibitory effect exerted by CM-CA, we conducted transcriptome sequencing (RNA sequencing) on differently treated tumors. As anticipated, findings confirmed expression alterations of the Wnt/β-catenin pathway in response to CA and CM-CA treatments. (**Figure 4A**) Encouragingly, in the case where the differences between the treated groups compared to the control group were both significant, the enrichment of inhibitory signatures was more pronounced in the CM-CA treated group. Subsequent analysis revealed a distinct down-regulation of genes functioning in cell proliferation and apoptosis associated with the Wnt/β-catenin signaling in the CM-CA group (42–44), further substantiating the superior inhibitory capacity of CM-CA over CA in targeting this pathway. (**Figure 4B**) In tumor cells, the hyperactive Wnt/β-catenin pathway plays a crucial role in regulating cell proliferation. The gene set enrichment analysis (GSEA) revealed notable suppressions of cell cycle related processes (cell cycle, cell cycle mitotic and cell cycle checkpoints) in CM-CA-treated tumor sample, contrasting with the limited effects of CA treatment. (**Figure 4C**) These findings collectively indicate that CM-CA exhibited enhanced Wnt/β-catenin pathway inhibition compared to CA, resulting in the suppression of tumor cell proliferation, which was consistent with observations at the animal and tissue levels.

Given the considerable heterogeneity observed in LUAD tumors, it is not uncommon for certain patients to exhibit inadequate intratumoral immune infiltration, which could potentially contribute to their resistance to conventional therapeutic approaches (1, 45). Motivated by the association between hyperactive Wnt/β-catenin signaling and the development of tumor immunosuppressive microenvironment, we were eager to investigate the potential of CM-CA in augmenting tumor immune responses. Through the utilization of mMCP-counter to quantify immune cell infiltration in tumor tissue, we were pleasantly astonished to discover a substantial increase in CM-CA treated sample compared with the others, particularly T cells as the main participants in the tumor immune response. (**Figure 4D**) Further analysis conducted through GSEA unveiled an enrichment of boosting signals in T cell activation involved in immune response in both samples treated with CA and CM-CA, especially the promoting trend observed in CM-CA-treated group was more conspicuous. (**Figure 4E**) This trend was further substantiated by estimation of tumor-infiltrating immune and stromal cells using ImmuCellAI-mouse. (**Figure 4F**) Remarkably, the CM-CA group exhibited a higher infiltration score and greater T cell infiltration compared to the other two groups. In summary, the aforementioned analyses demonstrate the efficacy of CM-CA in improving tumor immune infiltration while achieving potent Wnt/β-catenin pathway inhibition, leading to an augmented tumor immune response.

### 2.4 CM-CA possesses a favorable *in vivo* biosafety profile

**Figure 5:**
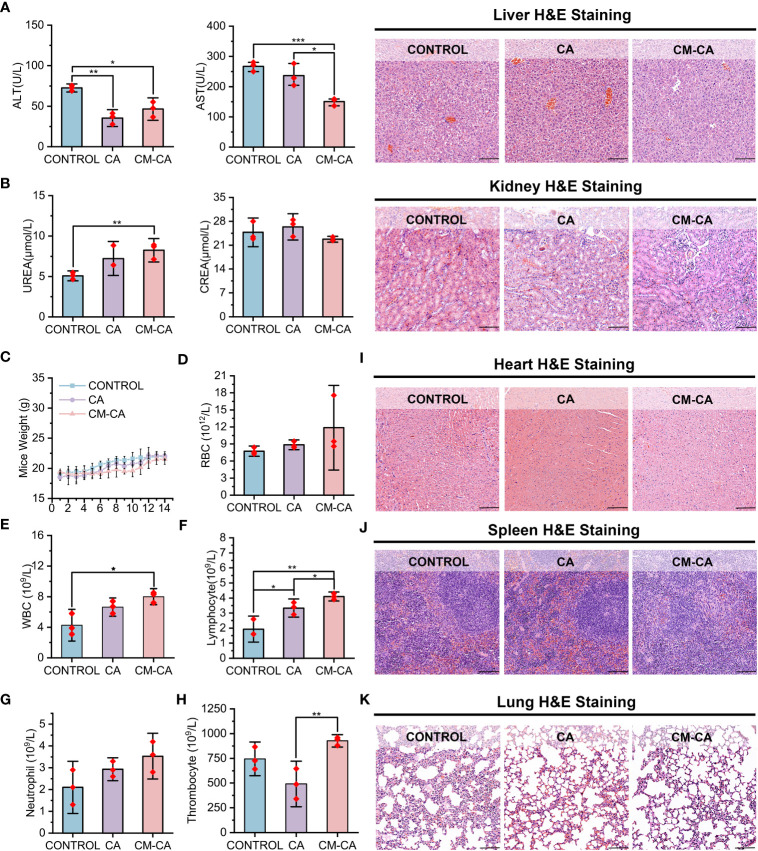
Biosafety profile of CM-CA. **(A)** Aspartate transaminase (AST), alanine aminotransferase (ALT) and H&E staining of liver pathological sections were used to determine the hepatotoxicity of CM-CA. (scale bar: 100 μm). **(B)** Blood urea nitrogen (UREA), creatinine (CREA) and H&E staining of kidney pathological sections were used to determine nephrotoxicity of CM-CA. (scale bar: 100 μm) **(C)** Body weights of mice in different groups during 14-day administration. **(D–H)** Measurement of red blood cell (RBC) **(D)**, white blood cell (WBC) **(E)**, lymphocyte **(F)**, neutrophil **(G)** and thrombocyte **(H)** in blood of mice. **(I–K)** H&E staining of pathological sections from mice heart **(I)**, spleen **(J)** and lung **(K)** after demonstrated treatments. (scale bar: 100 μm) The statistical difference was determined by t test. *, p<0.05; **, p<0.01; ***, p<0.001.

CA, a Wnt pathway small molecule inhibitor specifically targeting β-catenin/BCL9 protein-protein interaction (PPI), promotes the degradation of β-catenin and inhibits the activation of downstream target genes by perturbing β-catenin secondary structures near the binding site, inducing a conformational change in β-catenin and blocking the interaction with BCL9 (31, 46). However, the poor solubility of CA molecules in the water-based solution environment of the body poses challenges for its application. Additionally, there are concerns among researchers regarding the potential accumulation of CA at non-tumor sites when administered *in vivo* (22, 32, 41). CM-CA developed in this work is intended to employ CA as an active component for intracellular modulation of β-catenin/BCL9 PPI, with the anticipation of mitigating the systemic toxicity of CA by enhancing its tumor-targeting capability. Consequently, we investigated the condition of organs following the administration of control, CA and CM-CA treatment. As depicted in **Figure 5A**, concerning the liver function indicators, the levels of alanine transaminase (ALT) and aspartate aminotransferase (AST) remained within the normal range in mice from all three treatment groups, and there was no unusual disparity beyond the normal range between the CM-CA treatment group and the control group. The H&E staining results of the liver sections revealed no abnormal sites or intergroup differences, indicating the absence of significant liver damage in mice subjected to CM-CA treatment. Additionally, the kidney, being a vital metabolic organ in mice, exhibited no abnormally significant variations in serum urea (UREA) and creatinine (CREA) levels between the treatment and Control groups, suggesting that the renal filtration function remained unimpaired. (**Figure 5B**) Furthermore, the H&E staining results of the kidneys did not reveal any pathological abnormalities. It is worth mentioning that the tumor-bearing mice did not present any abnormal reduction in body weight during treatment, and there was no abnormal pattern of weight decrease or increase in the treatment groups when compared to the control group. (**Figure 5C**) The blood tests revealed that the levels of red blood cells (RBC), white blood cells (WBC), lymphocytes, neutrophils, and thrombocytes in all three groups of mice fell within the normal range, indicating that CM-CA treatment did not induce hematologic toxicity (**Figures 5D–H** and **Supplementary Figure S6**). The H&E staining images of the heart, spleen, and lungs, as presented in **Figures 5I–K**, also demonstrated the absence of any abnormalities resulting from CM-CA treatment. All of the above results indicate that our biomimetic Wnt suppressor does not cause systemic damage to vital organs and exerts its anti-tumor effects in a biologically safe manner.

## 3 Discussion

Non-small cell lung cancer accounts for up to 85% of lung cancers, and its predominant subtype is lung adenocarcinoma, which constitutes approximately 40% of all the cases (4, 5). Extensive research conducted over the past three decades has emphasized the significant involvement of Wnt/β-catenin signaling in lung cancer development, leading to numerous investigations exploring the potential of Wnt inhibition as an anti-tumor strategy (23). Currently developed Wnt inhibitors, including small molecules, peptides, and antibodies, face challenges associated with the implementation: small molecules exhibit a relatively high level of toxicity; peptides are prone to degradation and present difficulties in direct cellular utilization; antibodies pose complexities in production and their *in vivo* retention time remains unsatisfactory (47–50). To reconcile the intended acting mechanism of inhibitors with their practical application *in vivo*, researchers employ synthetic constituents like polyethylene glycol (PEG) to encapsulate inhibitors, thereby rendering them inconspicuous to the immune system and enabling their targeted recognition and utilization by tumor cells (51). Nevertheless, these synthetic components yield only modest extension of blood circulation, while the packaging procedure is intricate and beset with concerns pertaining to adverse immune reactions and toxicity (52, 53). As for target selection of Wnt signaling suppression, β-catenin presents as a promising candidate in lung cancer treatment. The applicability of specific targeted drugs is restricted due to multiple oncogenic mutations present frequently in NSCLC patients, and the ones without target driven gene mutations cannot benefit from it. Conversely, β-catenin is expressed in over half of NSCLCs and possesses rare variations, making inhibitors that target intracellular β-catenin advantageous in terms of both generalizability and efficacy (7, 54, 55). The interaction between β-catenin and BCL9 plays a crucial role in β-catenin-mediated transcription of downstream target genes in the nucleus, serving as a pivotal step in the activation of the Wnt pathway. Additionally, this binding exhibits a relatively substantial binding area and a moderate binding capacity, making it inherently advantageous for the development of inhibitors (47). CA, a small molecule derived from natural substances, was discovered to exhibit binding affinity towards the H1 helix of β-catenin in close proximity to BCL9 binding site, consequently disrupting the stability of β-catenin and facilitating its oligomerization and subsequent degradation (31, 56). This compound emerged as a highly promising therapeutic candidate for impeding the interaction between β-catenin and BCL9. Nevertheless, its advancement into clinical studies has been impeded by unresolved concerns regarding systemic toxicity (32, 57). CM-CA was deliberately designed with the aforementioned considerations in order to effectively and safely suppress the central component of Wnt pathway. (**Figure 1**) Additionally, it accomplishes tumor targeting with biosafety by encapsulating homologous tumor cell membranes. The presence of distinct antigens on the outer layer of cancer cells plays a significant role in facilitating the adherence of homotypic cancer cells to tumor tissues. The encapsulation of homotypic cancer cell membranes enables the effective enrichment of camouflaged nanoparticles at tumor sites, thereby enabling active targeted drug delivery (53, 58). This design features clear components and a straightforward synthesis process, circumventing toxicity concerns associated with superfluous modifications, as well as preventing a substantial increase in synthesis costs. Examined by LLC Lewis homograft model of LUAD, it was observed that CM-CA, possessed nearly twice the tumor growth inhibition value of CA, exhibited a greater capacity to suppress tumor growth compared to CA when administered at equal doses. (**Figure 3**) Analyses of immunohistochemical images and RNA sequencing revealed that CM-CA not only effectively targeted β-catenin to suppress Wnt/β-catenin signaling pathway, but also impede tumor cell proliferation and enhanced tumor immune infiltration, bolstering the immune response against tumors. (**Figures 3**, **4**) This implies that the biomimetic design strategy of Wnt suppressor, which appears to be straightforward yet efficient, is viable for *in vivo* implementation. In this particular context, it is important to highlight that there is a need for further reduction in the complexity of cell membrane encapsulation process, as well as an increase in the percentage of intact membrane-encapsulated particles, which is crucial for the successful identification and utilization of homologous tumors (53, 59).

Another potential application of CM-CA is to aid in addressing resistance to primary therapies for lung cancer. Among the current first-line treatments, chemotherapy and radiotherapy are widely used, and the activation of Wnt/β-catenin is associated with their resistance (27, 60). While molecularly targeted therapies that inhibit cancer driver genes have shown efficacy in patients with specific mutation types, they are often hindered by inevitable recurrence (61). Immunotherapy has emerged as a valuable treatment for patients diagnosed with driver-negative NSCLC, yet is plagued by poor response rates due to inferior immune infiltration and immunosuppressive microenvironment (62). Clinical data analysis has revealed a correlation between elevated β-catenin activity and inadequate immune infiltration in LUAD, leading to a less favorable prognosis. (**Figure 2**) This further supports the understanding that the aberrantly activated Wnt/β-catenin pathway is an important contributor to immunosuppression (42, 63). Promisingly, researches have been undertaken to incorporate Wnt/β-catenin inhibition into the established treating approaches, investigating the potential for combination therapy (22, 32, 64, 65). Actually, in an attempt to tackle the challenging immune milieu encountered in LUAD therapy, we endeavored to devise a universally applicable approach by amalgamating the β-catenin disruptor CA with the lewis lung cancer cell membrane to accomplish active targeting by means of recognizing and adhering to tumor cell membranes of the identical cancerous cells, thereby generating bionic particle CM-CA. In this work, CM-CA not only achieves the goal of disrupting intracellular β-catenin/BCL9 interaction in tumor cells, addressing the endogenous cause of insufficient immune infiltration, but also may function as a reservoir of tumor-associated antigens owing to its tumor cell mimicking properties, eliciting a heightened anti-tumor immune response (66). Our data indicate that compared to control or CA treatment, CM-CA treatment resulted in a higher abundance of immune cell infiltration and up-regulation of immune response-related pathways in tumor tissues. (**Figure 4**) This suggests that CM-CA has the potential to be used in conjunction with immunotherapy and other molecular targeted therapies. In recent times, there has been a surge in research focusing on the crucial regulatory function of the Wnt/β-catenin pathway in determining the fate and behavior of T cells (67–69). Consequently, it may be recommended that tumor-targeted Wnt inhibitors, such as CM-CA, be incorporated into conventional treatment protocols to manipulate the immune milieu and augment therapeutic effectiveness. However, further preclinical experiments are required to substantiate this proposition. In conclusion, this tumor-specific biomimetic Wnt pathway suppressor not only enhances the tumor immune response while ensuring biosafety, but also offers a promising strategy for prolonging the efficacy of other mainstream therapeutics.

## Data availability statement

Clinical data and mRNA expression matrix of LUAD samples analyzed in this work are obtained from TCGA database (https://portal.gdc.cancer.gov/) and the Gene Expression Omnibus (GEO, https://www.ncbi.nlm.nih.gov/geo/) database. The RNA sequencing datasets in this study are available in online repository: PRJNA1000099 [NCBI Sequence Read Archive]. All the methods and materials can be found in the Supplementary Material.

## Ethics statement

The animal study was approved by Medical Ethics Committee of Xi’an Jiaotong University. The study was conducted in accordance with the local legislation and institutional requirements.

## Author contributions

RG: Data curation, Formal Analysis, Investigation, Writing – original draft. XZ: Conceptualization, Investigation, Software, Writing – original draft. AJ: Investigation, Methodology, Validation, Writing – original draft. WH: Conceptualization, Project administration, Supervision, Writing – review & editing. TL: Conceptualization, Funding acquisition, Resources, Supervision, Validation, Visualization, Writing – original draft.
