# Supplementary material for: Modulating β-catenin/BCL9 interaction with cell-membrane-camouflaged carnosic acid to inhibit Wnt pathway and enhance tumor immune response
Source: Front Immunol. 2023 Oct 9;14:1274223. doi: 10.3389/fimmu.2023.1274223 (PMC10594212; doi:10.3389/fimmu.2023.1274223)
Supplement: Supplementary file 1 [file DataSheet_1.docx]

Supplementary Material

# Material and method

## Synthesis and physicochemical property check of CM-CA

To separate the cell membrane, LLC cells were harvested and suspended in a protease inhibitor-containing digestion buffer. They were then pulverized by intermittent sonication at 30% power for 2 minutes and centrifuged at 3200 g, 4℃ for 5 minutes. Next centrifuged the resulting supernatant at 10000 g, 4℃ for 20 minutes. Finally, the supernatant was continued to be centrifuged at 100000 g for 1h to obtain the cell membrane precipitate.

To synthesized CM-CA, we first used DMSO to dissolve CA at a concentration of 2mg/20μL, then diluted to 2mg/10mL by PBS. Next, CM-CA was obtained through filter by extruding encapsulation of CA and CM. The pellet's morphology was examined by transmission electron microscopy (TEM). A portion of CM-CA was positioned onto a carbon-coated copper grid, then imaging through TEM. CM-CA (1 mL) was loaded to dynamic light scattering (DLS) (Malvern Zetasizer Nano ZS system) to measure the hydrodynamic size distribution and the zeta potential. 50 μL CA (0.2mg/mL) and 50 μL centrifuged supernatant of CM-CA (7000 rpm, 5min) was respectively loaded to reverse phase high performance liquid chromatography (HPLC) to measure the encapsulation efficiency.

## LLC Cell culture

The LLC cell line was acquired from the cell bank of the Chinese Academy of Sciences (Shanghai, China) and preserved in DMEM medium. A 10% FBS media with 100 U/ml penicillin and 100 μg/ml streptomycin added was used to culture cells at 37°C in 5% CO_2_.

## Mouse study

The mice utilized in this study were procured from the Experimental Animal Center of Xi'an Jiaotong University. These mice were reared under controlled conditions, devoid of any specific pathogens, and provided with standard feed and regular light/dark cycles. All animal experimentation procedures adhered to the established guidelines and were approved by the Medical Ethics Committee of Xi'an Jiaotong University (approval number: 2020-277).

## Establishment of tumor model

Cell particles are suspended in sterile PBS through a centrifuge. LLC cells (8 × 10^5^ cells/site) were implanted under the buttock skin of C57BL/6 mice, aged between 5-6 weeks. When the average volume of the tumor reached ~50mm^3^, the mice were randomly divided into different groups (5 mice in each group) and treatment began. We randomly divided the mice into PBS control group, CA group (2mg/kg), and CM-CA group (2mg/kg), and administered them every other day through the tail vein, for a total of 7 cycles. The length and width of tumors are measured using a caliper, and the volume of tumors is calculated via: tumor volume (V)=length × width^2^/2.

We assessed the potential toxicity by monitoring the body weight of all mice during treatment and measuring hematologic and organ function indicators after 14 days of treatment. Mice were subjected to blood collection, then performed blood measurements as well as renal and liver function assays. When the experiment terminated, the mice were euthanized. For histological examination, tumor tissue was fixed with formaldehyde, dehydrated, cut into 4μm thick slices, and chemically stained with hematoxylin and eosin (H&E) or subjected to other immunohistochemical assays.

## H&E and immunohistochemistry (IHC) staining

The tumor tissue and major organs were dissected and immersed in a formaldehyde solution. Then pack it in paraffin and divide it into 4 μm thick. Then, in the conventional methods of histopathology, the slices were stained with hematoxylin and eosin (H&E). In addition, expression of Ki67 (Antibody from Protein, USA; 1:400) was detected through routine immunohistochemistry staining, and β-catenin (Antibody from Proteintech, USA; 1:1000) was detected in the tumor sections. Tumor section examination was performed through Pannoramic Desk scanning and measurement.

An immunostaining intensity (I) score ranging from 0 to 3 was used to assess staining intensity: 0, no staining; l, weak staining; 2, moderate staining; 3, intense staining. The immunostaining area (A) was evaluated using a numeric score ranging from 1 to 4 according to the intensity: 1, positive area <10%; 2, positive area=10-50%; 3, positive area=50-90%; 4, positive area >90%. IHC score was calculated as the intensity score (I) multiplied by the staining area score (A) (IHC score = I × A).

## Transcriptome analyses

The NEBNext® Ultra RNA Library Prep Kit for Illumina® (NEB England BioLabs) was utilized for the preparation of an RNA sequencing library. The fragmented and randomly primed 2 × 150 bp paired-end libraries were subsequently analyzed using the Next Illumina HiSeq X Ten platform. Heat maps and gene expression enrichment analysis were produced from using Qlucore Omics Explorer 3.2, while pathway analysis was conducted with Ingenuity Pathway Analysis (IPA) software. Two RNA-sequencing data-based methods were employed to estimate tumor-infiltrating immune and stromal cells in mice, including mMCP-counter (1) and ImmuCellAI-mouse (2). Heatmap plot was employed to visualize the immune and stromal cells infiltration level in each group of mice.

## Data Acquisition

We collected clinical and mRNA expression profiles of patients with lung adenocarcinoma (LUAD) from the Cancer Genome Atlas (TCGA, https://portal.gdc.cancer.gov/). Additionally, RNA-sequencing data and corresponding survival data from three series, namely GSE30219 (3), GSE11969 (4, 5), and GSE3141 (6), were downloaded from the Gene Expression Omnibus (GEO, https://www.ncbi.nlm.nih.gov/geo/) for further survival analysis. Furthermore, to estimate immune cell infiltration in patients with LUAD from the TCGA cohort, we utilized multiple algorithms from TIMER2.0 (http://timer.comp-genomics.org/timer/) (7).

## The association between CTNNB1 mRNA expression and clinical prognosis of patients with LUAD

The four aforementioned LUAD datasets were employed to explore the prognostic relevance of CTNNB1 in LUAD. Patients with LUAD were stratified into high and low CTNNB1 groups based on a predefined cut-off value determined using the "surv_cutpoint" function from the "survminer" package in R software. Kaplan-Meier survival curves were constructed to compare disease-free survival (DFS) or overall survival (OS) between the high and low CTNNB1 groups. The statistical difference was assessed using the log-rank test.

## The relationship between CTNNB1 mRNA expression and the infiltration level of CD8^+^ T cells and Tregs in the TCGA-LUAD cohort

The "corrplot" package in R was utilized to explore the correlation between CTNNB1 mRNA expression and the infiltration levels of CD8+ T cells and Tregs in the TCGA-LUAD cohort. Additionally, the correlation between CTNNB1 mRNA expression and the infiltration levels of CD8^+^ T cells estimated by CIBERSORT (8) and EPIC (9), as well as the infiltration levels of Tregs estimated by QUANTISEQ (10) and XCELL (11), were further visualized using correlation scatter plots.

## Gene Set Enrichment Analysis between CTNNB1^High^ and CTNNB1^Low^ samples in the TCGA-LUAD cohort

Gene Set Enrichment Analysis (GSEA) was conducted to identify the significantly altered signaling pathways or biological processes between CTNNB1^High^ and CTNNB1^Low^ samples. CTNNB1^High^ samples are referred as the mRNA expression level of CTNNB1 is greater than the top 75% of its expression level. Otherwise, they will be determined as CTNNB1^Low^ samples. Two reference gene sets, namely "h.all.v2023.1.Hs.symbols.gmt" and "c2.cp.kegg.v2023.1.Hs.symbols.gmt", were utilized for GSEA analysis. Significantly enriched terms with a P value < 0.05 and an adjusted P value < 0.25 were considered as indicative of altered signaling pathways or biological processes. The GSEA analysis was performed using R software, specifically employing the "clusterProfiler" and "enrichplot" packages.

## Statistical analysis

Statistical analyses were conducted utilizing bilateral student's t-test. Results were considered significant when calculated p value was less than 0.05. The data was presented as mean ± standard deviation. The statistical analysis outcomes are depicted in following form: *, p<0.05; **, p<0,01; ***, p<0001.

# Supplementary Figures


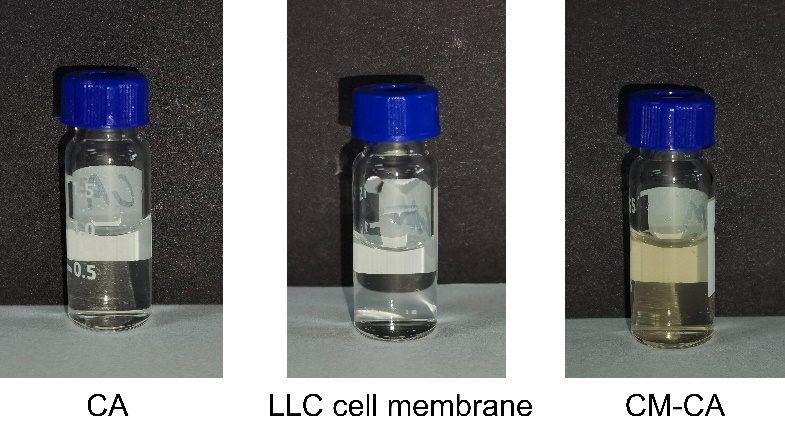


**Supplementary Figure 1.** Representative sample image of CA, LLC cell membrane and CM-CA (0.2mg/mL).


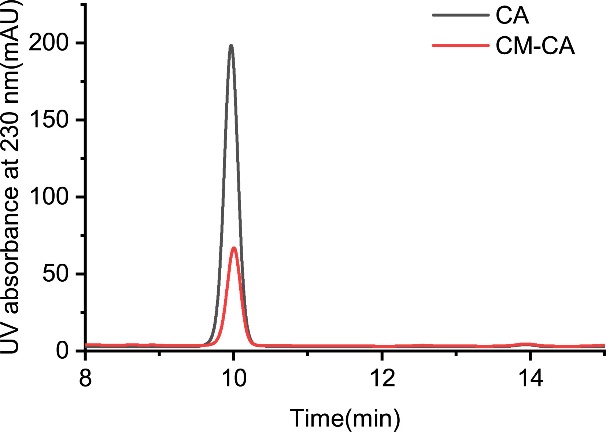


**Supplementary Figure 2.** UV absorbance at 230 nm of CA and CM-CA measured for encapsulation efficiency by HPLC. CA loading in the nanoparticle CM-CA was calculated to be 68.5%.


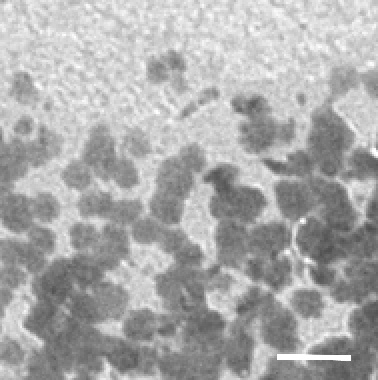


**Supplementary Figure 3.** Representative TEM image of CM-CA. (scale bar: 500 nm)

**
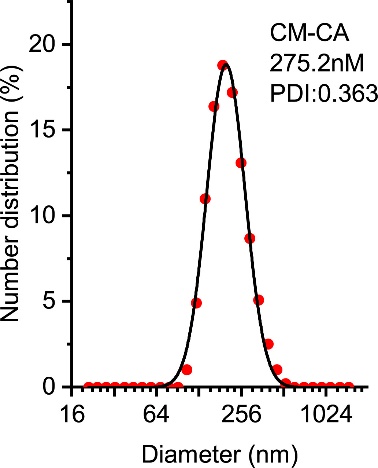
**

**Supplementary Figure 4.** Size distribution by number of CM-CA.


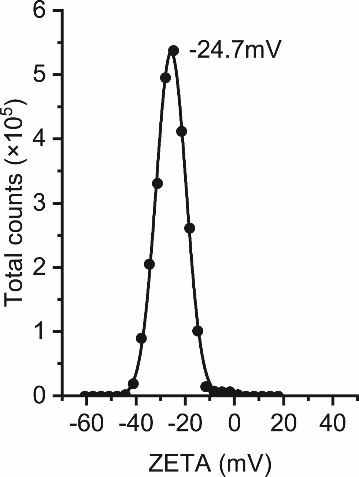


**Supplementary Figure 5.** Zeta potential of CM-CA measured in PBS at pH 7.4.


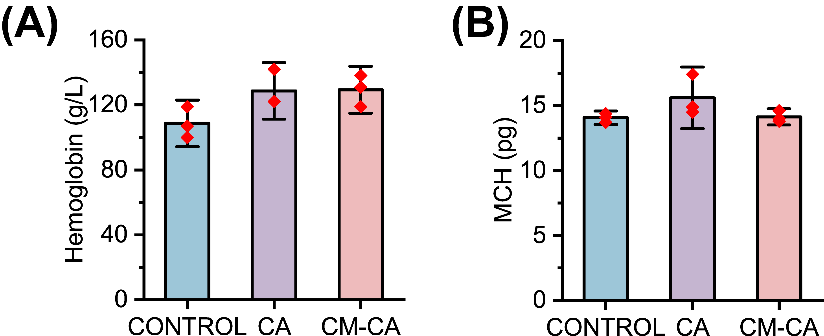


**Supplementary Figure 6.** Measurement of hemoglobin and mean corpuscular hemoglobin (MCH) of mice blood after indicated treatments.

**Reference**

1. Petitprez F, Levy S, Sun CM, Meylan M, Linhard C, Becht E, et al. The murine Microenvironment Cell Population counter method to estimate abundance of tissue-infiltrating immune and stromal cell populations in murine samples using gene expression. Genome Med. 2020;12(1):86.

2. Miao YR, Xia M, Luo M, Luo T, Yang M, Guo AY. ImmuCellAI-mouse: a tool for comprehensive prediction of mouse immune cell abundance and immune microenvironment depiction. Bioinformatics. 2022;38(3):785-91.

3. Rousseaux S, Debernardi A, Jacquiau B, Vitte AL, Vesin A, Nagy-Mignotte H, et al. Ectopic activation of germline and placental genes identifies aggressive metastasis-prone lung cancers. Sci Transl Med. 2013;5(186):186ra66.

4. Matsuyama Y, Suzuki M, Arima C, Huang QM, Tomida S, Takeuchi T, et al. Proteasomal non-catalytic subunit PSMD2 as a potential therapeutic target in association with various clinicopathologic features in lung adenocarcinomas. Mol Carcinog. 2011;50(4):301-9.

5. Takeuchi T, Tomida S, Yatabe Y, Kosaka T, Osada H, Yanagisawa K, et al. Expression profile-defined classification of lung adenocarcinoma shows close relationship with underlying major genetic changes and clinicopathologic behaviors. J Clin Oncol. 2006;24(11):1679-88.

6. Bild AH, Yao G, Chang JT, Wang Q, Potti A, Chasse D, et al. Oncogenic pathway signatures in human cancers as a guide to targeted therapies. Nature. 2006;439(7074):353-7.

7. Sturm G, Finotello F, Petitprez F, Zhang JD, Baumbach J, Fridman WH, et al. Comprehensive evaluation of transcriptome-based cell-type quantification methods for immuno-oncology. Bioinformatics. 2019;35(14):i436-i45.

8. Newman AM, Liu CL, Green MR, Gentles AJ, Feng W, Xu Y, et al. Robust enumeration of cell subsets from tissue expression profiles. Nat Methods. 2015;12(5):453-7.

9. Racle J, de Jonge K, Baumgaertner P, Speiser DE, Gfeller D. Simultaneous enumeration of cancer and immune cell types from bulk tumor gene expression data. Elife. 2017;6.

10. Finotello F, Mayer C, Plattner C, Laschober G, Rieder D, Hackl H, et al. Molecular and pharmacological modulators of the tumor immune contexture revealed by deconvolution of RNA-seq data. Genome Med. 2019;11(1):34.

11. Aran D, Hu Z, Butte AJ. xCell: digitally portraying the tissue cellular heterogeneity landscape. Genome Biol. 2017;18(1):220.
